# Adrenaline modulates the global transcriptional profile of *Salmonella *revealing a role in the antimicrobial peptide and oxidative stress resistance responses

**DOI:** 10.1186/1471-2164-9-458

**Published:** 2008-10-06

**Authors:** MH Karavolos, H Spencer, DM Bulmer, A Thompson, K Winzer, P Williams, JCD Hinton, CM Anjam Khan

**Affiliations:** 1 Institute for Cell and Molecular Biosciences, The Medical School, University of Newcastle, Newcastle NE2 4HH, UK; 2 Institute of Food Research, Norwich Research Park, Colney, Norwich NR4 7UA, UK; 3 School for Health and Medicine, Division of Biomedical and Life Sciences, Lancaster University, Lancaster, LA1 4YQ, UK; 4 Institute of Infection Immunity and Inflammation, University Park, University of Nottingham, Nottingham NG7 2RD, UK

## Abstract

**Background:**

The successful interaction of bacterial pathogens with host tissues requires the sensing of specific chemical and physical cues. The human gut contains a huge number of neurons involved in the secretion and sensing of a class of neuroendocrine hormones called catecholamines. Recently, in *Escherichia coli *O157:H7, the catecholamines adrenaline and noradrenaline were shown to act synergistically with a bacterial quorum sensing molecule, autoinducer 3 (AI-3), to affect bacterial virulence and motility. We wished to investigate the impact of adrenaline on the biology of *Salmonella *spp.

**Results:**

We have determined the effect of adrenaline on the transcriptome of the gut pathogen *Salmonella enterica *serovar Typhimurium. Addition of adrenaline led to an induction of key metal transport systems within 30 minutes of treatment. The oxidative stress responses employing manganese internalisation were also elicited. Cells lacking the key oxidative stress regulator OxyR showed reduced survival in the presence of adrenaline and complete restoration of growth upon addition of manganese. A significant reduction in the expression of the *pmrHFIJKLM *antimicrobial peptide resistance operon reduced the ability of *Salmonella *to survive polymyxin B following addition of adrenaline. Notably, both phenotypes were reversed by the addition of the β-adrenergic blocker propranolol. Our data suggest that the BasSR two component signal transduction system is the likely adrenaline sensor mediating the antimicrobial peptide response.

**Conclusion:**

*Salmonella *are able to sense adrenaline and downregulate the antimicrobial peptide resistance *pmr *locus through the BasSR two component signalling system. Through iron transport, adrenaline may affect the oxidative stress balance of the cell requiring OxyR for normal growth. Both adrenaline effects can be inhibited by the addition of the β-adrenergic blocker propranolol. Adrenaline sensing may provide an environmental cue for the induction of the *Salmonella *stress response in anticipation of imminent host-derived oxidative stress. However, adrenaline may also serve in favour of the host defences by lowering antimicrobial peptide resistance and hence documenting for the first time such a function for a hormone.

## Background

Bacterial pathogens can sense a variety of physical and chemical niche-specific cues enabling them to physiologically adapt and modulate virulence to survive and cause disease. To enable successful host-pathogen interactions it is increasingly recognised that bacteria must also respond to a diverse range of host effector molecules. The term "microbial endocrinology" was first used to describe the interactions of microbes with the neuroendocrine environment of their host [1]. Catecholamine hormones like adrenaline and noradrenaline are released in the bloodstream and are involved in the regulation of a wide variety of host physiological processes. Current data suggests that catecholamines can induce DNA damage via production of hydroxyl radicals in the presence of iron [2]. More recently, adrenaline was implicated in the production of hydroxyl radicals in rat hepatocytes via an adrenoreceptor-mediated mechanism [3].

There is evidence that non-neural cells like peripheral human T lymphocytes contain and are able to synthesize catecholamines from normal precursors in physiologic concentrations [4,5]. Recently, bacterial lipopolysaccharide has been shown to induce production and release of adrenaline and noradrenaline by macrophages and neutrophils [6]. It was therefore suggested that the phagocytic system represents a diffusely expressed adrenergic organ [6].

Both adrenaline and noradrenaline are present in the gastrointestinal system where they mediate normal gut physiology [7]. During infection, plasma levels of catecholamines rise in an increase previously associated with the onset of infection [8]. There is evidence to suggest that general stress can alter levels of these hormones in the gut and could act as an environmental cue for pathogens [8,9].

Indeed, catecholamines have been shown to induce both Gram negative and Gram positive bacterial growth via the provision of iron [10-15]. Noradrenaline affects production of the K99 pilus adhesin of enterotoxigenic *Escherichia coli *and also Shiga toxin in *E. coli *O157:H7 thus influencing the virulence fitness of these pathogens [16,17].

Although catecholamines represent a eukaryotic cell signal to mediate a concerted organ function, bacteria utilise a different form of communication mediated by small molecules termed "autoinducers" in a process called "quorum sensing" [18-20]. Briefly, bacteria produce and sense autoinducers (AIs) in a concentration-dependent fashion. Upon achievement of a critical concentration of autoinducer, a signal is generated to regulate processes such as bioluminescence, antibiotic biosynthesis, plasmid conjugation, biofilm formation, DNA uptake competence, sporulation, and virulence [21-23]. Recently, a novel autoinducer, AI-3, produced by *E. coli *and other Gram negative bacteria was shown to act in synergy with adrenaline and noradrenaline to regulate *E. coli *genes involved in motility and virulence independently of enterobactin-mediated iron transport [24]. Furthermore, α adrenergic antagonists were able to block these interactions suggesting sensory transduction through common receptors [25].

In this report we dissect the global effects of adrenaline on the *Salmonella enterica *serovar Typhimurium (S. Typhimurium) transcriptome. Our data show that approximately 0.6% of the transcriptome of the pathogen is significantly regulated by adrenaline. Most of the genes affected represent those involved in transport but we also see alterations in genes encoding proteins of unknown functions. We also notice changes in levels of regulators and signal transduction genes.

The major feature of the *S*. Typhimurium adrenaline response is the upregulation of genes involved in metal homeostasis and oxidative stress. Prompted by the transcriptomic data we investigated the expression of the manganese superoxide dismutase (*sodA*), and the regulators of iron homeostasis (*fur*) and oxidative stress (*oxyR*). Our evidence suggests that adrenaline provides an environmental cue to alert *S*. Typhimurium against impending macrophage-derived peroxide stress as shown by the reduced ability of *S*. Typhimurium lacking OxyR to survive in the presence of adrenaline.

Furthermore we identified a downregulation of the *pmrHFIJKLM *operon which encodes a well characterised lipid A-modification system that provides resistance to the cationic antimicrobial peptide polymyxin B. We investigated the expression of the *pmr *locus and suggest adrenaline-mediated reduction in antimicrobial peptide resistance is mediated by the BasSR two component signal transduction system.

The fact that adrenaline provides an environmental cue that alerts the bacterial defences against oxidative stress as well as acting in favour of the host by inducing a reduction in bacterial antimicrobial peptide resistance is a unique combination. This finding represents a novel insight concerning the role of hormones in pathogen-host interactions.

## Methods

### Bacterial Strains, Plasmids, and Growth Conditions

*S. enterica *serovar Typhimurium strains and plasmids are shown in Table 1. Strains were grown overnight in 5 ml LB broth and 25 μl of the overnight culture were used to inoculate 25 ml of LB in a 250 ml conical flask at 37°C, 200 rpm. After 3.5 h growth (OD_600_~1.0), adrenaline was added at a final concentration of 50 μM from a fresh stock (100 mM). Incubation was continued for an additional 30 minutes, cultures were harvested by centrifugation and RNA extracted as described below. General physiological, molecular biological and protein manipulations were performed according to standard laboratory protocols [26,27]. Antibiotics were added to cultures at designated concentrations [27]. The gene content of strains used in transcriptomic experiments was confirmed by genomic indexing [28].

**Table 1:** Strains plasmids and primers used in this study

Strains		
Name	**Genotype**	**Reference**

**SL1344**	Parent strain	[73]
**SL1344*oxyR***	SL1344 Δ*oxyR*	This work
**SL1344*fur***	SL1344 Δ*fur*	This work
**SL1344*sodA***	SL1344 Δ*sodA*	This work
**SL1344*basS***	SL1344 Δ*basS*	This work
**SL1344pA**	SL1344 (pMK1*lux*-P_sodA_)	This work
**SL1344pM**	SL1344 (pMK1*lux*-P_pmrH_)	This work

Plasmids		

Name	**Description**	**Reference**

**pBR322**	Cloning vector	[74]
**pSB377**	*luxCDABE *reporter operon	[75]
**pMK1*lux***	pBR322 with *luxCDABE *operon and MCS	This work
**pMK1*lux*-P**_**sodA**_	pMK1*lux *with P_sodA _cloned as 5'EcoRI-3'BamHI fragment	This work
**pMK1*lux*-P**_**pmrH**_	pMK1*lux *with P_pmrH _cloned as 5'EcoRI-3'BamHI fragment	This work

Primers		

Name	**Sequence**	**Comment**

**sodA5**	GCG**GAATTC**ATCAACAGGCG	cloning
**sodA3**	GCG**GGATCC**ATTATTGTCGAGC	cloning
**pmr5**	CGC**GAATTC**GCGAAATAGCGTTTG	cloning
**pmr3**	CGC**GGATCC**ATTGAAAGCCGCTTTTC	cloning
**pmr q5**	ATGTCGGACTTTTTGCCTTTC	qPCR
**pmr q3**	ATATTGATTGCCAGTTAGCC	qPCR
**sodA q5**	ATGAGTTATACACTGCCATC	qPCR
**sodA q3**	GCAAACTCAGGCAGGTTTTC	qPCR
**fhuA q5**	ATGGCGCGTCTTAAAACTGC	qPCR
**fhuA q3**	GCGGCAGGCGCTGCGGTTAC	qPCR
**invF q5**	ATGTCATTTTCTGAAAGCCG	qPCR
**invF q3**	AATGCCAGTAATTTGCTGAG	qPCR
**entE q5**	ATGCGTATACCTTTCACCCG	qPCR
**entE q3**	CTGAATGCGCGCTCGCCTTC	qPCR
**bas5**	CGCACGGTTCGCGGGTTTGG	λ-red
**bas3**	GTAGTGTGCTGATTGTCAGC	λ-red
**bas-P1**	CTACATGCTGGTTGCCACTGAGGAAAGCTAAGTGAGCCTGGTGTAGGCTGGAGCTGCTTC	λ-red
**bas-P4**	AGTTTTATCTATGTGTGGGTCACGACGTATTAAACGCCTGATTCCGGGGATCCGTCGACC	λ-red
**fur5**	AGTGCAATTTCTGTCACTTC	λ-red
**fur3**	CAGGAAAGAGGAGGATATAA	λ-red
**fur-P1**	TCTAATGAAGTGAATCGTTTAGCAACAGGACAGATTCCGCGTGTAGGCTGGAGCTGCTTC	λ-red
**fur-P4**	AAAAGCCAACCGGGCGGTTGGCTCTTCGAAAGATTTACACATTCCGGGGATCCGTCGACC	λ-red
**oxy5**	TAATCGTTCATTGCTATGCT	λ-red
**oxy3**	AACACCACCTTTAACTACCC	λ-red
**oxy-P1**	ACCTATCGCCATGAACTATCGTGGCGACGGAGGATGAATAGTGTAGGCTGGAGCTGCTTC	λ-red
**oxy-P4**	TCGGGTTGCGGCGTTGAACGGCTTAAACCGCCTGTTTTAAATTCCGGGGATCCGTCGACC	λ-red

Restriction endonuclease sites are in bold.

### Transcriptomics experimental design and methodology

RNA was isolated from cultures according to protocols described on the IFR microarray web site . Briefly, two OD units of culture was fixed by incubation on ice with a 1/5 culture volume of 5% phenol and 95% ethanol to immediately stop RNA transcription or degradation. Cultures were centrifuged at 4,000 rpm for 10 minutes, and the resulting pellets were frozen at -80°C. RNA was subsequently isolated using an SV Total RNA system (Promega) following the protocols provided by the manufacturer. The quality of the RNA was verified using an Agilent 2100 Bioanalyzer (Agilent), and the quantity was determined with an ND-1000 spectrophotometer (Nanodrop). Microarray hybridisation and scanning were performed at the Institute of Food Research, (IFR) Norwich as described previously [28,29] and according to protocols described on the IFR microarray web site . Briefly, RNA samples (16 μg) from three biological replicates and two technical replicates were labelled with Cy5-dCTP and hybridized to the IFR SALSA microarrays. Cy3-dCTP-labeled *S*. Typhimurium genomic DNA was used as a common reference in an indirect comparison type experimental design [30]. The IFR SALSA microarrays comprise 5080 genes from 5 different serovars of *Salmonella *.

### Transcriptomics data analysis validation and *in silico *informatics

Microarrays were scanned and fluorescence intensities quantified using GenePix Pro software, version 6.0 (Axon Instruments, Inc.). Microarray features showing a reference signal lower than background plus 2 standard deviations were discarded. Unequal dye incorporation was compensated by median centering (see ). Transcriptomic data from adrenaline containing LB cultures was normalised to data from LB cultures without adrenaline and significant differences at P ≤ 0.05 were determined using a parametric-based statistical test by adjusting the individual P-value with the Benjamini and Hochberg false discovery rate multiple test correction [31]. All expression data for genes discussed in the text have passed this filter and are therefore statistically significant. These tests are features of the GeneSpring™ 7.2 (Silicon Genetics) microarray analysis software package which was used for both data visualisation and analysis. The analysis was based on statistically significant differences displaying greater than 1.5 fold changes between LB cultures with and without adrenaline. In general transcriptomic data are filtered to only include equal to or greater than 2-fold differences, however less than 2-fold changes can also be biologically significant [32,33].

Validation of microarray transcriptomic data was performed by quantitative RT-PCR (qPCR) analysis using the Qiagen QuantiTect SYBR Green system and a Roche Lightcycler 480. Primers used for validation analysis are listed in Table 1. Motif searches on protein sequences were carried out using "SMART" [34] and "PFAM" [35]. For protein homologies we used BLAST .

### Construction of expression vectors

The *luxCDABE *operon was amplified by PCR from pSB377 using primers *lux*5 and *lux*3 (Table 1). The PCR product containing an engineered multiple cloning site (MCS; *Eco*RI, *Sac*I, *Kpn*I, *Bam*HI, *Xba*I, *Sna*BI) upstream of the *lux *operon was then *Eco*RI/*Eag*I digested and ligated to *Eco*RI/*Eag*I-cut pBR322 giving rise to pMK1*lux*. Promoters were cloned using the *Eco*RI and *Bam*HI restriction sites of the MCS of pMK1*lux*. For a list of promoter primers and plasmid constructs see Table 1.

Expression from promoter-*lux *transcriptional expression vectors was evaluated by growing *S*. Typhimurium containing the specific expression vector in 25 ml of LB in a 250 ml conical flask at 37°C, 200 rpm. After 3.5 h of growth adrenaline (50 μM), propranolol (500 μM), or water were added and incubation was continued for a further 30 minutes. Samples (200 μl) were harvested, and the optical density and luminescence were determined with a Tecan Infinite200 spectrophotometer. Experiments were repeated at least three times.

### Adrenaline stress assay

For determination of stress resistance during exposure to different adrenaline concentrations, bacteria were grown overnight in 5 ml LB broth and 25 μl of the overnight culture was used to inoculate 25 ml of LB in a 250 ml conical flask at 37°C, 200 rpm. After 3.5 hours growth (OD_600_~1.0), the following, or combinations were added; adrenaline (50 μM), propranolol (500 μM), manganese (5 mM) or water. Incubation was continued for an additional 3 h, and serial dilutions were plated out on LB plates. Experiments were repeated at least three times and data are presented as survival numbers with standard error bars.

### Measurement of total Fe

This was done as described by Velayudhan *et al*., (2007), with some modifications [36]. Bacteria were grown overnight in LB (5 ml), then subcultured in 25 ml fresh LB and grown at 37°C, 200 rpm for 2 h. Adrenaline (50 μM), propranolol (500 μM) or H_2_O was added and incubation was continued for an additional 4 h. Cells were harvested, washed twice in 25 ml of 10 mM EDTA, pH 8.0 and twice in 25 ml of analytical grade water (< 0.01p.p.m., Sigma). The OD_600 _and the volume of the cell suspension in the last wash were recorded. The final cell pellet was weighed and then solubilised by resuspending in 0.75 ml of 30% ultra-pure nitric acid at 80°C for 16 h. The volume was increased to 7 ml with water before analysis by inductively coupled plasma atomic emission spectroscopy (ICP-AES) using a UNICAM 701 Series Emission Spectrometer (Chemical and Materials Analysis, Newcastle University). Five replicas per condition were carried out. Standard error bars are shown.

### Antimicrobial peptide assay

Cells were evaluated for their ability to resist killing by the antimicrobial peptide polymyxin B. This was done as described by Fields *et al*. (1989), with some modifications [37]. Bacteria exposed to adrenaline (50 μM), propranolol (500 μM) or water were aliquoted in a 96-well plate at a concentration of 2 × 10^4 ^to 5 × 10^4 ^bacteria per well, in 50 μl of a solution containing 0.5% tryptone and 0.5% sodium chloride. A 100 μl volume of antimicrobial peptide was added (polymyxin B, 0.15 μg ml^-1^; Sigma) and the plate was incubated at 37°C, 150 rpm for 1 h. Samples were collected and viable counts performed by plating out different dilutions on LB plates. Data are presented as colony forming units and represent the average of three independent experiments.

Array Express; Accession Number E-MEXP-1738.

## Results and discussion

### Microarray Analysis of *Salmonella *Adrenaline Transcriptome

During infection bacteria come into contact with a wide range of host-derived molecules ranging from very small molecular weight compounds to peptides and proteins. Adrenaline is produced by the host in specialised organ tissues [38]. Recently it was shown that phagocytes and polymorphonuclear cells are capable of *de novo *production of catecholamines, and when exposed to lipopolysaccharide *in vitro *they release noradrenaline and adrenaline [6]. These findings stimulated our interest in investigating the effects of adrenaline on *S. enterica *serovar Typhimurium. We used adrenaline at the concentration of 50 μM to reflect experiments previously performed by others [24] and sampled at 30 minutes post-addition.

**Figure 1:**
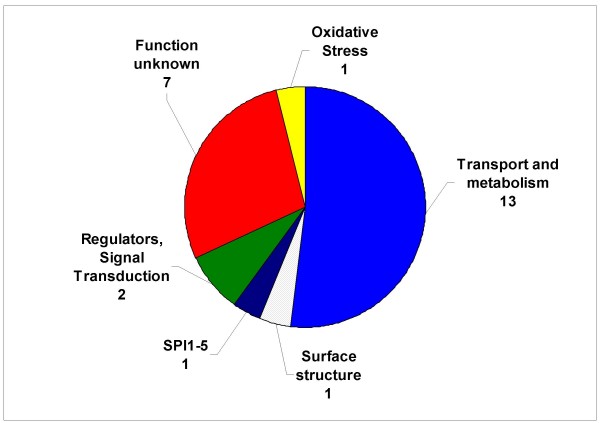
**The *S*. Typhimurium adrenaline-regulated transcriptome**. Pie chart displaying number of genes in selected categories significantly altered upon addition of adrenaline (50 μM). A detailed list of the genes can be found in Table 2.

The transcriptomic data showed that the addition of adrenaline leads to a significant regulation (P ≤ 0.05) of 25 genes with alterations ranging from 0.4 to 2.3 fold (Table 2). Interestingly, more that 52% of the adrenaline-regulated genes were involved in transport and metabolism and approximately a third encoded proteins of unknown function (Fig 1).

**Table 2:** Adrenaline regulated genes of *S*. Typhimurium

**A**			
**Adrenaline Upregulated genes**			

**KEGG annotation**	**Product**		**Fold change**

**Transport and metabolism**			

STM0192, *fhuC*	ATP-binding component of hydroxymate-dependent iron transport		**2.3**
STM3159, *exbB*	uptake of enterochelin; *tonB*-dependent uptake of B colicins		**2.2**
STM0191, *fhuA*	outer membrane protein receptor for ferrichrome		**2.0**
STM3158, *exbD*	uptake of enterochelin; *tonB*-dependent uptake of B colicins		**2.0**
STM0596,*entE*	2,3-dihydroxybenzoate-AMP ligase		**1.8**
STM3506, *feoB*	ferrous iron transport protein B		**1.8**
STM3505,*feoA*	ferrous iron transport protein A		**1.8**
STM2861, *sitA*	*fur *regulated *Salmonella *Mn transporter		**1.6**
STM2862, *sitB*	*fur *regulated *Salmonella *Mn transporter		**1.5**
**Oxidative Stress**			

STM4055, *sodA*	superoxide dismutase		**1.9**
**Function unknown**			

STM1728, *yciG*	putative cytoplasmic protein		**1.8**
STM2263, *yojI*	putative ABC-type multidrug/protein/lipid transport system		**1.7**
STM1586	putative periplasmic protein, similar to *E. coli *putative receptor		**1.7**
STM1729,*yciF*	putative cytoplasmic protein		**1.7**

**B**			

**Adrenaline Downregulated genes**			

**KEGG annotation**	**Product**		**Fold change**

**Transport and metabolism**			

STM2299,*yfbG *(*pmrI*)	transformylase		**0.4**
STM1935, *ftn*	cytoplasmic ferritin		**0.4**
STM2297, *yfbE *(*pmrH*)	4-amino-4-deoxy-L-arabinose LPS-modifying enzyme		**0.5**
STM2298, *pmrF*	glycosyl transferase		**0.5**
**Surface structure**			

STM1176, *flgD*	flagellar hook capping protein		**0.7**
**SPI1-5**			

STM2899, *invF*	invasion protein		**0.6**
**Regulators, Signal Transduction**			

STM2301, *pqaB *(*pmrK*)	polymyxin B resistance		**0.6**
STM3216	putative methyl-accepting chemotaxis protein II, aspartate sensor receptor		**0.7**
**Function unknown**			

STM1936, *yecH*	putative cytoplasmic protein		**0.5**
STM4293, *yjdB*	putative integral membrane protein		**0.6**
STM2300, *pmrJ*	cytoplasmic protein		**0.6**

**C**			

**Microarray Validation**			

		**Fold change**	
		
**KEGG annotation**	**Product**	**Microarrays**	**qPCR**

STM0191, *fhuA*	outer membrane protein receptor for ferrichrome	**2.03**	**2.46**
STM0596, *entE*	2,3-dihydroxybenzoate-AMP ligase	**1.80**	**1.29**
STM4055, *sodA*	superoxide dismutase	**1.90**	**1.18**
STM2297, *yfbE *(*pmrH*)	4-amino-4-deoxy-L-arabinose LPS-modifying enzyme	**0.45**	**0.45**
STM2899, *invF*	invasion protein	**0.64**	**0.57**

The majority of the upregulated genes encoded components involved in iron transport, microcin, and oxidative stress resistance. Most of the genes displaying decreased expression levels belonged to the BasSR-regulated *pmrHFIJKLM *operon which encodes a lipid A modification system [39,40].

Among the downregulated genes was *flgD*, encoding a flagellar basal body rod modification protein, and *invF*, involved in *Salmonella *Pathogenicity Island 1 (SPI-1) mediated Type 3 secretion (T3S). We did not observe a significant difference in *S*. Typhimurium motility or the SPI-1 mediated T3S secreted protein profile during exposure to adrenaline (data not shown). However in *E. coli*, studies performed to assess the role of catecholamines on the transcriptome have revealed significant changes in both motility and T3S genes [41,42]. This may reflect important biological differences between the two organisms under the conditions tested. In agreement with our observations these studies also identified upregulation of iron transport genes.

The transcriptomic results were validated by qPCR (Table 2) and also by the use of luminescent transcriptional reporters and a range of phenotypic screens. We constructed promoter transcriptional fusions to investigate the oxidative stress response using *sodA *(upregulated) and the antimicrobial peptide resistance *pmr *operon (downregulated) as described below.

### Transport systems affected by adrenaline in *Salmonella*

**Figure 2:**
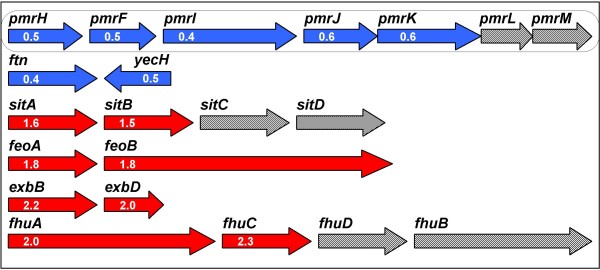
**Diagrammatic representation of major operons affected by adrenaline**. Chromosomal organisation of genes showing a significant transcriptional change upon addition of adrenaline (50 μM). Respective fold change values in relation to the untreated control are displayed within the individual gene. Unaffected genes within an operon are represented by skewed lines.

The majority of adrenaline-regulated genes are involved in metal transport, uptake of siderophores and microcins (Table 2 and Fig. 2). *fhuA *and *fhuC *encode components of the hydroxamate-dependent iron transport system in *Salmonella *spp. and are also the receptors for microcin J25 [43]. Microcin J25 stimulates the production of reactive oxygen species such as superoxide ion (O_2_^-^) in bacterial cells, leading to damage via perturbation of the membrane respiratory chain [44]. In *E. coli *the ferric hydroxamate uptake receptor FhuA transports siderophores in a TonB-dependent manner [45,46]. The *exbBD *system, participating in the TonB-dependent uptake of microcin J25 in *E. coli *and responsible for enterochelin and B colicin uptake [47] is also significantly upregulated. Induction of such systems may provide a valuable insight into the way adrenaline affects bacterial physiology to modulate host-pathogen interactions during infection.

Two additional systems involved in manganese, *sitAB *and iron transport, *feoAB*, [48] are also upregulated by adrenaline. The *sitABCD *locus encodes an important transporter of manganese and iron which is required for resistance to H_2_O_2 _and for full virulence of *S*. Typhimurium in animals [49-51]. SitA is also required for *Salmonella *spp. virulence in macrophages by facilitating manganese transport [52]. Bacterial accumulation of manganese forms the basis for an alternative catalytic detoxification of reactive oxygen species, the exact mechanism of which is not yet completely understood [53]. We hypothesise that intracellular manganese accumulation reflected an adrenaline-induced mechanism to aid pathogen survival. The downregulation of the *S*. Typhimurium *ftn *gene encoding a ferritin involved in iron storage [54], may mirror the perturbation in the general metal pool during exposure to adrenaline.

**Figure 3:**
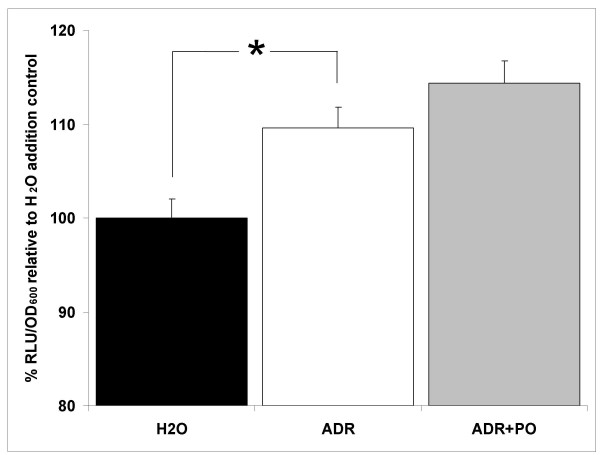
**Adrenaline affects expression of *sodA***. A luminescent reporter fusion system (pMK1*lux*-P_*sodA*_) was used to assess expression of the *sodA *gene during exposure to adrenaline. Exposure of SL1344pA to adrenaline (50 μM) for 30 minutes resulted in a significant (10%) increase in *sodA *expression (P ≤ 0.05). Addition of β-adrenergic blocker propranolol (500 μM) did not reverse the effect of adrenaline. Luminescence is expressed as a percentage of the water addition control in relative light units per culture optical density (RLU/OD_600_). Experiments were repeated at least three times. Asterisk indicates significant difference by the student t-test. Standard error bars are shown. Water, H_2_O; Adrenaline, ADR; Propranolol, PO.

The superoxide dismutase, *sodA*, gene is also significantly upregulated by adrenaline (Table 2). The *S*. Typhimurium manganese-cofactored superoxide dismutase (SodA) is involved in resistance to the early oxygen-dependent microbicidal mechanisms of phagocytes [55]. Using a luminescent *sodA *transcriptional reporter we observed a slight (10%) but significant (P ≤ 0.05) increase in *sodA *expression supporting the transcriptomic results and also highlighting the presence of increased oxidative stress by exposure to adrenaline (Fig. 3). The effect was not blocked by addition of β-adrenergic blocker propranolol (Fig. 3). We used a *S*. Typhimurium strain lacking *sodA *(SL1344*sodA*) to further characterise the importance of the superoxide dismutase in the response to adrenaline. We did not observe a significant change in the numbers of bacteria surviving exposure to 50 μM adrenaline when compared to the wild type SL1344, suggesting that *sodA *is not essential for survival during exposure to adrenaline (data not shown).

The above *S*. Typhimurium transcriptional signature suggests a dual role for adrenaline. On the one hand, by inducing iron uptake systems, it serves as a warning probing the bacteria to adjust their metal ion transport in such a way as to resist looming oxidative stress but, on the other hand, facilitating increased susceptibility to microcin assault by increasing microcin J25 receptor content.

### OxyR and not Fur is essential for survival during adrenaline exposure

**Figure 4:**
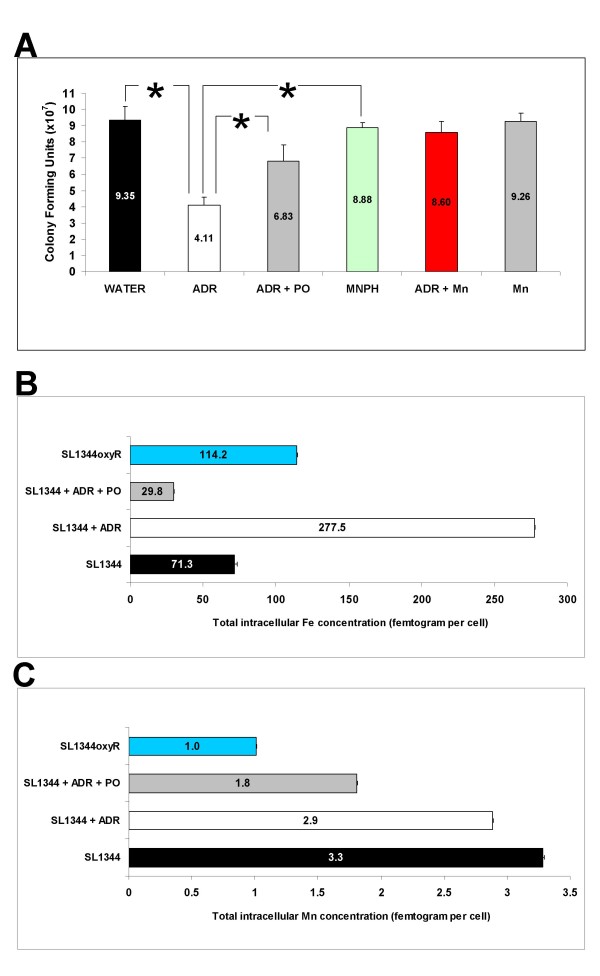
**OxyR and manganese are necessary for survival upon exposure to adrenaline**. The role of the major oxidative stress regulator OxyR in the ability of *Salmonella *spp. to survive exposure to various adrenaline concentrations was assessed. (**A**) Deletion of *oxyR *in SL1344*oxyR *results in a dramatic loss of viability when exposed to adrenaline (50 μM) for 30 minutes. The effect of adrenaline is significantly lessened by addition of propranolol (PO) at 500 μM. Addition of the adrenaline derivative metanephrine (50 μM) which cannot bind iron has no significant effect on cell viability. Addition of manganese (5 mM) also fully counteracts the growth inhibition observed by adrenaline while the metal by itself (Mn) does not affect survival. SL1344 viability is unaffected by adrenaline (data not shown). (**B**) Measurement of total cell iron indicating a significant 4-fold increase upon exposure of SL1344 to adrenaline, reduced levels upon simultaneous exposure to propranolol and adrenaline, and also significantly elevated levels in SL1344*oxyR*. (**C**) Measurement of total cell manganese indicating significantly reduced (3-fold) levels in SL1344*oxyR *and slightly reduced levels in SL1344 upon addition of adrenaline or simultaneous exposure to propranolol and adrenaline. Experiments were repeated at least three times. Asterisk indicates significant difference by the student t-test. Standard error bars are shown. Adrenaline, ADR; Propranolol, PO; Metanephrine, MNPH; Manganese, Mn.

Oxidative stress resistance in bacterial cells is mediated by enzymatic as well as non-enzymatic methods involving manganese [53,56]. OxyR is a positive regulator of a range of genes implicated in resistance to hydrogen peroxide [56,57]. Fur fine-tunes the regulation of iron homeostasis by controlling iron transport [56,58]. Having observed a significant upregulation of iron and manganese transporter genes by adrenaline, we examined the importance of *fur *and *oxyR *during *Salmonella *exposure to adrenaline. Exposure of SL1344 *fur *to 50 μM adrenaline had no effect on its ability to grow in rich growth media suggesting *fur*-mediated functions are not important in the adrenaline response (data not shown). However, SL1344*oxyR *survived significantly less in the presence of adrenaline when compared to wild type SL1344 with the phenotype being blockable by the addition of the β-adrenergic blocker propranolol (Fig. 4A). To test if the effect was due to the ability of adrenaline to bind iron we treated SL1344*oxyR *with metanephrine, a natural methylated metabolite of adrenaline which is unable to bind iron [59]. Addition of 50 μM metanephrine had no significant effect on the survival of SL1344*oxyR *supporting the role of adrenaline-bound iron in reducing the viability of the strain (Fig. 4A).

### Manganese rescues *oxyR *in the presence of adrenaline

We tested the ability of manganese to improve survival of SL1344*oxyR *treated with adrenaline by supplementing the growth medium with the metal. Addition of 5 mM manganese fully restored bacterial survival back to wild type levels in SL1344*oxyR *treated with 50 μM adrenaline (Fig. 4A). The importance of manganese in alleviating the oxidative stress effect in cells is related to its ability to reduce the effects of the Fenton reaction involving intracellular iron [53]. By a mechanism not fully elucidated yet, manganese acts as a natural free radical detoxifying agent reacting with superoxide and also hydrogen peroxide.

Measurement of the total metal ion concentration in cells treated with 50 μM adrenaline as well as in the *oxyR *strain supports the hypothesis that adrenaline induces oxidative stress by promoting an increase in the intracellular iron concentration (Fig. 4B). We observe a 4-fold increase in the total iron concentration of cells treated with 50 μM adrenaline when compared to the water-treated control (Fig. 4B). Furthermore, addition of β-adrenergic blocker propranolol blocks the adrenaline-mediated increase in the total iron concentration (Fig. 4B). SL1344*oxyR *has significantly increased total iron and reduced total manganese concentration when compared to SL1344 (Fig. 4B, C). This fact in conjunction with the adrenaline-induced increase in intracellular iron may explain the reduced viability of SL1344*oxyR *upon adrenaline treatment and subsequent rescuing of viability with manganese (Fig. 4A). However, the role of propranolol in rescuing SL1344*oxyR *during exposure to adrenaline may be independent of manganese. This is highlighted by the reduction (~2 fold) in total manganese levels upon exposure to propranolol.

Adrenaline may therefore induce oxidative stress via an OxyR-dependent pathway in a manner reversible by the β-adrenergic blocker propranolol and also by the non-enzymatic manganese-based oxidative stress detoxification system.

### Adrenaline reduces expression of the *pmr *locus and increases sensitivity to polymyxin B

Lipid A is a structural component of the lipopolysaccharide (LPS) in the outer membrane of Gram-negative bacteria and plays an important role in bacterial pathogenesis [60]. Polymyxin B, is a cationic antimicrobial peptide which binds to lipid A and damages the cell envelope [61]. Resistance to antimicrobial peptides has been shown to contribute to persistence of *S*. Typhimurium in a variety of niches ranging from the phagosomes within macrophages to the *C. elegans *intestine [62,63]. The *pmrHFIJKLM *operon encodes a set of proteins involved in lipopolysaccharide modification and resistance to the cationic antimicrobial polypeptide polymyxin B [64,65]. The resistance mechanism involves attachment of phosphoethanolamine and 4-amino-4-deoxy-L-arabinose moieties on lipid A reducing its net negative charge and limiting its interaction with polymyxin [39,66]. In *S*. Typhimurium, the *pmr *locus is under the control of the BasSR two component system [39,67].

**Figure 5:**
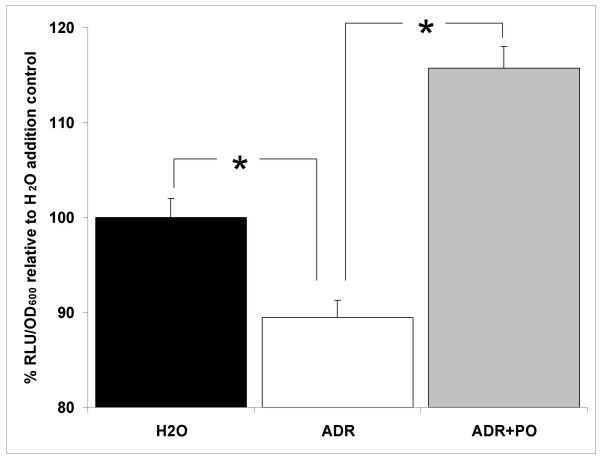
**Expression of the *pmr *locus is reduced by adrenaline**. Expression of the *pmrHFIJKLM *operon after a 30 minute exposure to adrenaline (50 μM) was assessed in SL1344pM by measuring luminescence per OD_600 _as described in "Methods". The significant transcriptional reduction (10%; P ≤ 0.05) in expression was fully reversed by addition of β-adrenergic blocker propranolol (500 μM). Luminescence is expressed as a percentage of the water addition control in relative light units per culture optical density (RLU/OD_600_). Experiments were repeated at least three times. Asterisk indicates significant difference by the student t-test. Standard error bars are shown. Water, H_2_O; Adrenaline, ADR; Propranolol, PO.

To further elucidate the effect of adrenaline on the *pmr *operon we constructed a transcriptional reporter fusion driving expression of the *luxCDABE *operon under the control of the *pmr *promoter. Addition of adrenaline significantly (P ≤ 0.05) reduced expression from the *pmr *promoter mirroring the array results (Fig. 5). The transcriptional downregulation of the *pmr *locus by adrenaline was fully reversible by the β-adrenergic blocker propranolol (Fig. 5).

**Figure 6:**
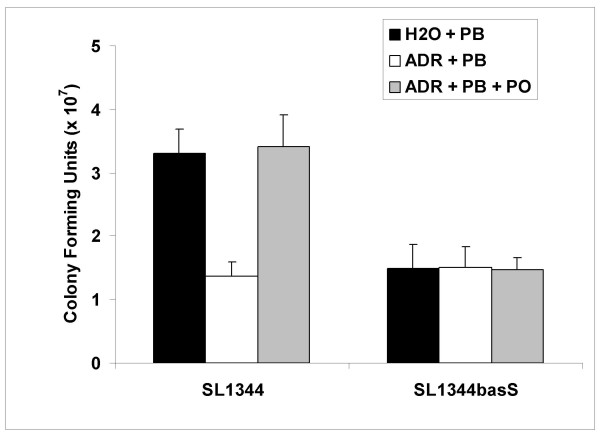
**Adrenaline modulates the ability of *Salmonella *to resist antimicrobial peptides**. We tested the effect of pre-exposure to adrenaline on the ability of *Salmonella *to resist the antimicrobial peptide polymyxin B. Addition of adrenaline (50 μM) significantly reduced *Salmonella *survival during exposure to polymyxin B (0.15 μg ml^-1^). This was fully reversed by the β-adrenergic blocker propranolol (500 μM). Sensitivity levels of the *basS *mutant (SL1344*basS*) to polymyxin B were very similar to those of the adrenaline-treated SL1344. Reversal of polymyxin B sensitivity by propranolol is dependent on the presence of *basS*. Experiments were repeated at least three times. Asterisk indicates significant difference by the student t-test. Standard error bars are shown. Water, H_2_O; Adrenaline, ADR; Polymyxin B, PB; Propranolol, PO.

We hypothesised that a reduction in expression of the *pmr *locus would lead to increased sensitivity to the antimicrobial peptide polymyxin B. We tested the effect of adrenaline on the ability of *Salmonella *to resist polymyxin B by incubating *Salmonella *exposed to water or adrenaline to the antimicrobial peptide as detailed in "Methods and Materials". Pre-treatment of *Salmonella *with 50 μM adrenaline resulted in a significant reduction in bacterial survival during exposure to polymyxin B when compared to the water treated control (Fig. 6). The adrenaline-induced reduction in the ability of *Salmonella *to resist polymyxin B was also fully reversible by the β-adrenergic blocker propranolol (Fig. 5).

The above data show a direct and reversible reduction of bacterial antimicrobial peptide resistance by a mammalian hormone and hence a novel "antibacterial" role for adrenaline. However, we note that *Salmonella *may have adapted to this negative effect of adrenaline within mammalian hosts by increasing Lipid A deacylation and palmitoylation, thus favouring survival via reduced TLR-4 receptor-based bacterial signalling [68,69].

### Adrenaline-induced sensitivity to polymyxin B may be mediated via the BasSR two component system

In enterohemorrhagic *E. coli *O157:H7 the QseBC two component system senses adrenaline and is required for full virulence in a rabbit animal model [70]. The *E. coli *response to adrenaline was shown to be blockable by an α-adrenergic antagonist [70]. In *S*. Typhimurium the BasSR two component system controls expression of the *pmr *locus and is implicated in the regulation of various other genes [39,40,71]. The identity at the amino acid level between the BasS and QseC sensor kinases is 31% (over 270 amino acids; BLAST). Based on this similarity and also on the observed effects of adrenaline on the *pmr *operon, we chose to further investigate the role of the sensory protein BasS in the mediation of the adrenaline response.

We constructed a *S*. Typhimurium SL1344 strain lacking the membrane sensor kinase BasS (SL1344*basS*) and tested its ability to survive polymyxin B in the presence or absence of adrenaline. Survival of SL1344*basS *was significantly reduced (P ≤ 0.05) in the presence of polymyxin B due to downregulation of the *pmr *locus as previously published [67] (Fig. 6). Levels of polymyxin B resistance in water-treated SL1344*basS *were very similar to those observed in adrenaline-treated wild type SL1344. Furthermore, although addition of the β-adrenergic blocker propranolol significantly improved the survival of SL1344 to polymyxin B during exposure to adrenaline, survival of SL1344*basS *remained unaffected by the β-adrenergic blocker (Fig. 6).

The above data support the hypothesis that adrenaline exerts its effect on the *pmr *locus via the reversible interaction of the β-adrenergic blocker with the BasS membrane sensor in a manner similar to the interaction of adrenaline with QseC in *E. coli*. The low (31%) amino acid sequence identity between BasS and QseC may provide a clue as to why we observe β-blockage in *Salmonella *as opposed to α-blockage in *E. coli*.

## Conclusion

Bacterial-host communication is increasingly being recognised as important in determining the outcome of infection. It is clear that bacterial pathogens encounter a wide range of host milieus, within which they must survive to successfully colonise and cause disease. *Salmonella *can replicate and survive within the harsh environment of the macrophage [37,72]. Our transcriptomic approach has revealed the response of *Salmonella *to adrenaline highlighting its dual role in mediating host-bacterial interactions. Systemic or macrophage produced adrenaline may therefore regulate the fine balance between the host and *Salmonella *defence mechanisms, and impact upon the development of disease.

## Authors' contributions

MHK and CMAK conceived the study. All authors played a role in designing the laboratory experiments and analyzing the data. Microarray experiments were conducted by MHK and also AT. The microarray validations, reporter constructions, and phenotypic screens were conducted largely by MHK, with the exception of the following: HS constructed the *oxyR *and *fur *mutant strains and conducted the respective adrenaline sensitivity tests; DMB constructed the *basS *mutant. The research was coordinated by CMAK. MHK drafted the initial manuscript with important subsequent contributions and revisions from PW, KW, JCDH, and CMAK. All authors have read and approved the final manuscript.
